# Comparison of clinicopathologic characteristics and survival outcomes between invasive IPMN and invasive MCN: A population-based analysis

**DOI:** 10.3389/fonc.2022.899761

**Published:** 2022-07-29

**Authors:** Zhen Yang, Guangjun Shi

**Affiliations:** Department of Hepatopancreatobiliary Surgery, Qingdao Municipal Hospital, Qingdao University, Qingdao, China

**Keywords:** IPMN, MCN, clinical characteristic, survival, treatment

## Abstract

**Background:**

Intraductal papillary mucinous neoplasm (IPMN) and mucinous cystic neoplasm (MCN) are two main histological subtypes of pancreatic cystic neoplasms with rapidly increasing incidence recently. The natural histories, treatment patterns, and survival outcomes of invasive IPMN and invasive MCN have not been well explored.

**Methods:**

Patients with a diagnosis of invasive IPMN and invasive MCN in the SEER database from 2000 through 2018 were retrospectively identified. Multivariable Cox regression analysis was conducted to determine the independent risk factors associated with overall survival (OS). Subgroup analyses of survival outcomes for invasive IPMN and invasive MCN were conducted. The OS for invasive IPMN was compared between patients who underwent surgery alone and those who received surgery plus chemotherapy by propensity score matching (PSM).

**Results:**

A total of 2,505 patients were included, of whom 2,300 were diagnosed with invasive IPMN and 205 were diagnosed with invasive MCN. Half of the invasive IPMN (48.4%) and three-quarters of the invasive MCN (76.1%) patients were female. Of all patients, both the OS and cancer-specific survival were significantly better in the invasive MCN cohort compared to the invasive IPMN cohort. In subgroup analyses, while invasive MCN experienced better OS compared to invasive IPMN in the subgroups of patients with local–regional disease, the survival advantages disappeared in patients at a distant stage. In addition, surgery plus chemotherapy in invasive IPMN patients was associated with significantly better survival compared to surgery alone after PSM.

**Conclusion:**

We examined the demographic and clinical characteristics between invasive IPMN and invasive MCN patients using a large-population-based analysis. Although the OS is significantly better for invasive MCN *versus* invasive IPMN, the difference disappeared in patients with distant disease. A combination of surgery and chemotherapy in selected invasive IPMN patients could confer survival benefits compared to surgery alone.

## Introduction

Pancreatic cystic neoplasms (PCNs) are regarded as challenging entities as they can exhibit a disease spectrum from benign to malignant lesions (1). The incidence of incidentally discovered PCNs has been gradually rising worldwide over the last few decades due to the increased use of cross-sectional imaging (2, 3). Intraductal papillary mucinous neoplasm (IPMN) and mucinous cystic neoplasm (MCN) are two main histological subtypes which have been recognized as precursors of pancreatic cancer (4). IPMN is a mucin-producing clinical entity arising from the pancreatic duct system, while MCN is a distinctive subtype with the presence of ovarian-type stroma and almost exclusively occurs in middle-aged females (5, 6). The risk of progression to malignancy varies between IPMN and MCN (7). However, the natural history and malignant potentials were not fully understood yet. Given the divergent malignant potentials and increasing frequency of these two predominant lesions, it is crucial to differentiate between these various types and formulate clinical guidelines for decision-making. Regarding the variable clinical course and limited high-quality research on invasive IPMN and invasive MCN, there also remain ambiguities in distinction between these two diseases. Moreover, the management of pancreatic cysts remains controversial (8, 9). The European guidelines suggest conservative treatment for IPMN and MCN with less than 4 cm in size and in the absence of worrisome features (10), while the international and American guidelines recommend aggressive surgical resection for all MCNs and IPMN >3 cm (11, 12). Additionally, larger population-based analyses investigating the clinicopathologic features and survival outcomes are scarce.

Thus, the purpose of this current study was to determine and compare the demographic and clinical characteristics of invasive IPMN *versus* invasive MCN as well as evaluate the treatment strategies using a large population in the United States.

## Methods

Data regarding patients with invasive IPMN or invasive MCN between 2000 and 2018 were retrieved from the SEER database which represents about 30% of the population in the United States (13). The inclusion criteria were as follows: (1) patients with a histological confirmation of invasive IPMN or invasive MCN, (2) patients with invasive IPMN or invasive MCN labeled as the first and only primary tumor, and (3) patients with a follow-up duration of more than 1 month. Patients with missing information on survival status or treatment details were excluded. The parameters collected in our study included age at diagnosis (<70/≥70 years), gender (female/male), race (white/black/other), marital status (married/other), tumor size, tumor location (head/body and tail), tumor differentiation (well differentiated/poorly differentiated/unknown), lymph node status (positive/negative/unknown), tumor stage (localized/regional/distant), treatment details (surgery/chemotherapy/radiation), and survival data (time and status). The primary outcomes were overall survival (OS) and cancer-specific survival (CSS).

### Statistical analysis

Continuous variables were presented as mean ± standard deviation and compared using independent-samples *t*-test, while categorical variables were described as number (percentage) and compared using chi-square analysis. OS and CSS were calculated by the Kaplan–Meier method, and comparisons were conducted with log-rank test. Univariate and multivariate COX analyses were performed to identify independent risk factors associated with OS or CSS. Additionally, in order to compare the efficacy between surgery and surgery plus chemotherapy in patients with invasive IPMN, propensity score matching (PSM) analysis was utilized to balance the baseline characteristics and increase between group comparability (14, 15). A *p*-value <0.05 was considered statistically significant. All analyses were performed by SPSS, version 26.0 and R version 4.0.3.

## Results

During the study period, a total of 2,505 patients diagnosed with invasive IPMN (*N* = 2,300) or invasive MCN (*N* = 205) between 2000 and 2018 in the United States with complete data were identified and analyzed in our study according to the inclusion and exclusion criteria. In the invasive IPMN cohort, most of the cases were localized at the pancreatic head (62.7%), with single tumor (94.0%), and only 42.6% received surgery. While in the invasive MCN cohort, the vast majority of patients were female (76.1%), younger than 70 years old (67.3%), white (76.1%), with single tumor (90.7%), and more than 85% had a surgical resection performed. In addition, invasive MCNs were more commonly found in the pancreatic body and tail (68.3%). As for the tumor characteristics, the tumor diameters were 4.26 ± 3.52 and 6.87 ± 8.08 cm in the invasive IPMN and invasive MCN groups, respectively. The most common tumor stage at presentation was distant in patients with invasive IPMN while localized in patients with invasive MCN. Unknown regional node status in these two cohorts accounted for 54.8 and 23.4%, respectively. The more detailed demographic and treatment data are summarized in **Table 1**. As presented in **Table 1**, the variables including gender, age at diagnosis, tumor size, tumor location and number, tumor stage, regional node status, distant metastasis, and treatment modalities were significantly different between invasive IPMN and MCN cohorts.

**Table 1:** Baseline characteristics of IPMN and MCN patients.

Variables	All patients (*n* = 2,505)	IPMN (*n* = 2,300)	MCN (*n* = 205)	*P*-value
Gender				<**0.001**
Male	1,236 (49.3%)	1,187 (51.6%)	49 (23.9%)	
Female	1,269 (50.7%)	1,113 (48.4%)	156 (76.1%)	
Age (years)				**0.019**
<70	1,494 (59.6%)	1,356 (59.0%)	138 (67.3%)	
≥70	1,011 (40.4%)	944 (41.0%)	67 (32.7%)	
Race				0.197
Black	279 (11.1%)	254 (11.0%)	25 (12.2%)	
White	2,011 (80.3%)	1,855 (80.7%)	156 (76.1%)	
Other	215 (8.6%)	191 (8.3%)	24 (11.7%)	
Tumor size (cm), ± SD	4.47 ± 4.14	4.26 ± 3.52	6.87 ± 8.08	<**0.001**
Marital status				0.893
Married	1,514 (60.4%)	1,391 (60.5%)	123 (60.0%)	
Other	991 (39.6%)	909 (39.5%)	82 (40.0%)	
Location				<**0.001**
Head	1,507 (60.2%)	1,442 (62.7%)	65 (31.7%)	
Body/tail	998 (39.8%)	858 (37.3%)	140 (68.3%)	
Grade				0.138
Well differentiated	966 (38.6%)	874 (38.0%)	92 (44.9%)	
Poorly differentiated	354 (14.1%)	326 (14.2%)	28 (13.6%)	
Unknown	1,185 (47.3%)	1,100 (47.8%)	85 (41.5%)	
Tumor number				**0.042**
Single	2,354 (94.0%)	2,168 (94.3%)	186 (90.7%)	
Multiple	151 (6.0%)	132 (5.7%)	19 (9.3%)	
Tumor stage				<**0.001**
Localized	437 (17.4%)	339 (14.7%)	98 (47.8%)	
Regional	898 (35.8%)	827 (36.0%)	71 (34.6%)	
Distant	1,170 (46.8%)	1,134 (49.3%)	36 (17.6%)	
Regional nodes positive				<**0.001**
Yes	517 (20.6%)	478 (20.8%)	39 (19.0%)	
No	680 (27.2%)	562 (24.4%)	118 (57.6%)	
Unknown	1,308 (52.2%)	1,260 (54.8%)	48 (23.4%)	
Liver involvement	293 (11.7%)	292 (12.7%)	1 (0.5%)	<**0.001**
Lung involvement	173 (6.9%)	172 (7.5%)	1 (0.5%)	<**0.001**
Surgery				<**0.001**
Done	1,155 (46.1%)	979 (42.6%)	176 (85.9%)	
None	1,350 (53.9%)	1,321 (57.4%)	29 (14.1%)	
Radiation				0.566
Done	523 (20.9%)	477 (20.7%)	46 (22.4%)	
None	1,982 (79.1%)	1,823 (79.3%)	159 (77.6%)	
Chemotherapy				<**0.001**
Done	1,426 (56.9%)	1,350 (58.7%)	76 (37.1%)	
None	1,079 (43.1%)	950 (41.3%)	129 (62.9%)	

IPMN, intraductal papillary mucinous neoplasm; MCN, mucinous cystic neoplasm; SD, standard deviation. Bold indicates significance.

### Survival outcomes

**Figure 1:**
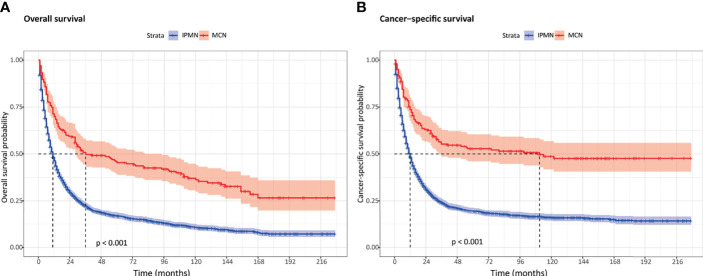
The overall survival and cancer-specific survival between invasive intraductal papillary mucinous neoplasm and invasive mucinous cystic neoplasm. **(A)** Overall survival. **(B)** Cancer-specific survival.

Of all patients in our study, both the OS and CSS were significantly better in the invasive MCN cohort compared to the invasive IPMN cohort (**Figure 1**). Among the patients with invasive IPMN, the 1-, 3-, and 5-year OS was 46.3, 22.2, and 16.6%, respectively. With respect to patients with invasive MCN, the 1-, 3-, and 5-year OS was 69.9, 49.7, and 45.3%, respectively. The median OS was 11 months for invasive IPMN and 36 months for invasive MCN. As for CSS, the survival probability at 1, 3, and 5 years was 48.3, 24.2, and 19.3% in the IPMN cohort, respectively, and 73.6, 55.2, and 52.4% in the MCN cohort, respectively. The median CSS was more favorable in the MCN group than in the IPMN group (**Table 2**).

**Table 2:** Survival outcomes of patients with IPMN and MCN.

Outcomes		IPMN (*n* = 2,300)	MCN (*n* = 205)	*P*-value
Overall survival	1 year	46.3%	69.9%	*P* < 0.001
	3 year	22.2%	49.7%	*P* < 0.001
	5 year	16.6%	45.3%	*P* < 0.001
	Median	11 months	36 months	*P* < 0.001
Cancer-specific survival	1 year	48.3%	73.6%	*P* < 0.001
	3 year	24.2%	55.2%	*P* < 0.001
	5 year	19.3%	52.4%	*P* < 0.001
	Median	12 months	111 months	*P* < 0.001

IPMN, intraductal papillary mucinous neoplasm; MCN, mucinous cystic neoplasm.

**Figure 2:**
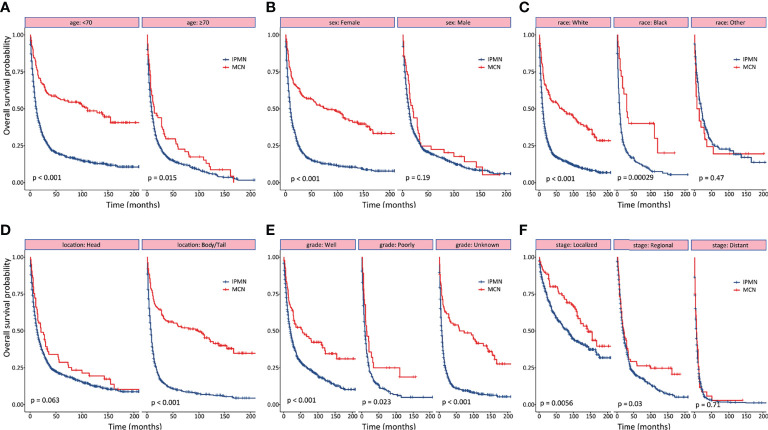
Subgroup analyses of overall survival between patients with invasive intraductal papillary mucinous neoplasm and invasive mucinous cystic neoplasm. **(A)** age. **(B)** gender. **(C)** race. **(D)** tumor location. **(E)** tumor differentiation. **(F)** tumor stage.

As shown in **Figure 2**, the survival outcomes in all prespecified subgroups were estimated and compared according to age at diagnosis, sex, race, tumor location, tumor grade, and tumor stage, respectively. Significant differences were observed for OS in the subgroup analysis except for male, of tumor located at the pancreatic head, and distant disease (**Figure 2**). The 1-, 3-, and 5-year OS and CSS probabilities at different stages are summarized in **Table 3**.

**Table 3:** Survival outcomes of patients with IPMN and MCN in different stages.

Outcomes		Localized		Regional		Distant	
		IPMN (*n* = 339)	MCN(*n* = 98)	IPMN (*n* = 827)	MCN(*n* = 71)	IPMN (*n* = 1,134)	MCN(*n* = 36)
Overall survival	1 year	82.4%	90.7%	62.6%	63.4%	23.8%	26.0%
	3 year	65.0%	79.7%	29.0%	30.7%	4.2%	5.8%
	5 year	56.2%	74.6%	19.4%	26.3%	2.5%	2.9%
	Median	NA	NA	18 months	20 months	6 months	6 months
Cancer-specific survival	1 year	83.5%	95.6%	64.8%	67.0%	25.5%	26.7%
	3 year	68.9%	88.2%	31.3%	34.5%	4.6%	6.0%
	5 year	63.0%	85.8%	22.2%	31.7%	2.9%	3.0%
	Median	NA	NA	20 months	23 months	6 months	7 months

IPMN, intraductal papillary mucinous neoplasm; MCN, mucinous cystic neoplasm.

### Treatment patterns and the relative survival outcomes in invasive IPMN and MCN patients

**Figure 3:**
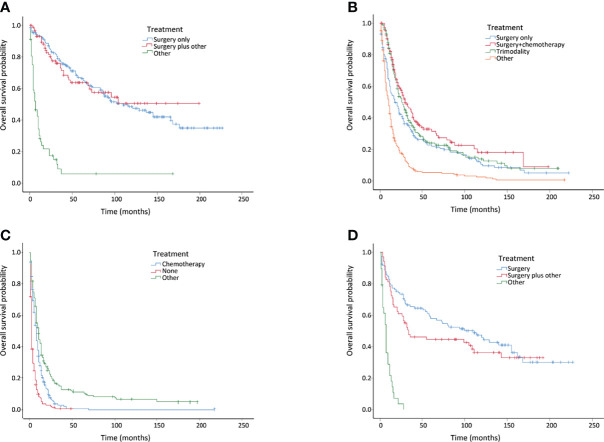
Kaplan–Meier curves of overall survival in invasive intraductal papillary mucinous neoplasm (IPMN) and invasive mucinous cystic neoplasm (MCN) patients with different treatments. **(A)** Invasive IPMNs with localized disease. **(B)** Invasive IPMNs with regional disease. **(C)** Invasive IPMNs with distant disease. **(D)** Entire invasive MCNs.

The treatment patterns of the different stages are summarized in **Table 4**. Surgery remained the main treatment option for patients with localized disease in the invasive IPMN cohort, whereas chemotherapy was more commonly performed in the distant disease. IPMN patients at a localized stage who only underwent surgery were found to associate with a similar overall survival compared to surgery plus other treatment, whereas surgery plus chemotherapy provided survival benefits in patients with regional disease (**Figures 3A, B**). However, patients with distant disease were associated with poor prognosis, and there were no effective treatments as yet (**Figure 3C**). In terms of the patients with invasive MCN, we found that surgery yielded comparable survival results compared to surgery plus other treatments (**Figure 3D**).

**Table 4:** Treatment patterns of IPMN and MCN patients in different stages.

Patterns	IPMN			MCN		
	Localized (*n* = 339)	Regional (*n* = 827)	Distant (*n* = 1,134)	Localized (*n* = 98)	Regional (*n* = 71)	Distant (*n* = 36)
Surgery only	192	188	37	69	33	5
Chemotherapy only	12	72	565	0	1	5
Radiation only	2	8	23	0	0	1
Surgery–chemotherapy	57	177	46	13	6	8
Trimodality	27	213	19	14	22	4
None of the three	30	79	368	2	5	12

IPMN, intraductal papillary mucinous neoplasm; MCN, mucinous cystic neoplasm.

### Analysis of risk factors for OS in patients with invasive IPMN or MCN

On multivariate analysis of patients with invasive IPMN (*n* = 2,300), age at diagnosis, race, marital status, tumor location, tumor grade, tumor stage, regional nodes status, liver involvement, surgery, and radiation were significant prognostic factors for OS. With respect to OS in patients with invasive MCN, age at diagnosis, tumor grade, tumor stage, and surgery were independent risk factors (**Table 5**).

**Table 5:** Univariate and multivariate analysis for overall survival in IPMN and MCN patients.

Variables	IPMN				MCN			
	Univariate analysis		Multivariate analysis		Univariate analysis		Multivariate analysis	
	HR (95% CI)	*P*-value	HR (95% CI)	*P*-value	HR (95% CI)	*P*-value	HR (95% CI)	*P*-value
Gender								
Male	Ref		Ref		Ref		Ref	
Female	0.89 (0.81–0.97)	**0.008**	1.01 (0.92–1.11)	0.830	2.07 (1.43–3.00)	<**0.001**	1.32 (0.87–1.99)	0.189
Age (years)								
<70	Ref		Ref		Ref		Ref	
≥70	1.30 (1.19–1.43)	<**0.001**	1.37 (1.25–1.50)	<**0.001**	2.70 (1.90–3.83)	<**0.001**	2.30 (1.58–3.36)	<**0.001**
Race								
White	Ref		Ref		Ref		Ref	
Black	1.22 (1.06–1.40)	**0.006**	1.16 (1.01–1.34)	**0.047**	1.24 (0.74–2.07)	0.424	1.46 (0.85–2.49)	0.168
Other	0.70 (0.58–0.83)	<**0.001**	0.73 (0.61–0.87)	<**0.001**	2.03 (1.24–3.33)	**0.005**	1.18 (0.69–2.02)	0.548
Tumor size (cm)	1.01 (1.00–1.01)	<**0.001**	1.00 (0.99–1.01)	0.315	1.01 (0.99–1.01)	0.568		
Marital status								
Married	Ref		Ref		Ref			
Other	1.24 (1.13–1.36)	<**0.001**	1.24 (1.13–1.36)	<**0.001**	0.95 (0.67–1.34)	0.759		
Location								
Head	Ref		Ref		Ref		Ref	
Body/tail	1.55 (1.41–1.70)	<**0.001**	1.19 (1.08–1.32)	**0.001**	0.55 (0.39–0.79)	**0.001**	0.81 (0.53–1.22)	0.314
Grade								
Well differentiated	Ref		Ref		Ref		Ref	
Poorly differentiated	1.69 (1.47–1.95)	<**0.001**	1.39 (1.21–1.60)	<**0.001**	1.78 (1.08–2.92)	**0.023**	1.71 (1.01–2.91)	**0.047**
Unknown	1.86 (1.69–2.06)	<**0.001**	1.09 (0.97–1.22)	0.143	1.04 (0.71–1.51)	0.854	0.87 (0.57–1.33)	0.526
Tumor number								
Single	Ref				Ref			
Multiple	1.48 (0.68–3.22)	0.318			0.68 (0.38–1.24)	0.212		
Tumor stage								
Localized	Ref		Ref		Ref		Ref	
Regional	2.52 (2.12–2.99)	<**0.001**	1.93 (1.59–2.34)	<**0.001**	2.78 (1.85–4.15)	<**0.001**	2.21 (1.40–3.50)	**0.001**
Distant	6.66 (5.62–7.89)	<**0.001**	2.48 (2.02–3.05)	<**0.001**	8.85 (5.46–14.33)	<**0.001**	5.40 (2.92–9.97)	<**0.001**
Regional nodes positive								
Yes	Ref		Ref		Ref		Ref	
No	2.36 (2.04–2.75)	<**0.001**	1.73 (1.47–2.05)	<**0.001**	2.58 (1.66–4.01)	<**0.001**	1.01 (0.61–1.69)	0.966
Unknown	5.38 (4.72–6.14)	<**0.001**	1.79 (1.47–2.18)	<**0.001**	3.15 (2.10–4.72)	<**0.001**	1.55 (0.90–2.65)	0.112
Liver involvement	2.98 (2.56–3.46)	<**0.001**	1.24 (1.06–1.46)	**0.009**	1.08 (0.86–1.37)	0.514		
Lung involvement	2.07 (1.73–2.47)	<**0.001**	0.943 (0.78–1.14)	0.536	1.08 (0.85–1.36)	0.532		
Surgery								
None	Ref		Ref		Ref		Ref	
Done	0.23 (0.21–0.26)	<**0.001**	0.43 (0.36–0.52)	<**0.001**	0.15 (0.10–0.24)	<**0.001**	0.43 (0.21–0.88)	**0.021**
Radiation								
None	Ref		Ref		Ref			
Done	0.76 (0.68–0.84)	<**0.001**	0.82 (0.73–0.92)	**0.001**	0.93 (0.62–1.39)	0.716		
Chemotherapy								
None	Ref				Ref			
Done	0.91 (0.83–1.01)	0.053			1.07 (0.75–1.53)	0.693		

IPMN, intraductal papillary mucinous neoplasm; MCN, mucinous cystic neoplasm; Ref, reference. Bold indicates significance.

### Comparison of therapeutic efficacy in patients with invasive IPMN after PSM

**Figure 4:**
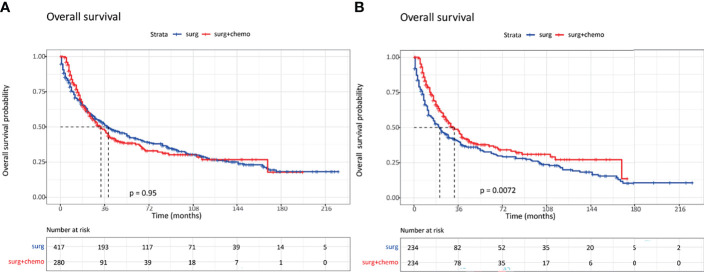
Kaplan–Meier curves of overall survival in invasive intraductal papillary mucinous neoplasm patients with surgery alone and surgery plus chemotherapy. **(A)** Survival curves in unmatched patients. **(B)** Survival curves in matched patients.

In order to examine the survival benefits of surgery plus chemotherapy in patients with invasive IPMN, we compared the therapeutic efficacy between surgery and surgery plus chemotherapy using the PSM method. Before PSM, age at diagnosis, tumor grade, and tumor stage were significantly different between the two cohorts. Surgery plus chemotherapy was more frequently performed in patients with older age (≥70), poorly differentiated tumor, and advanced stages. The overall survival was similar in the unmatched cohorts (**Figure 4A**). After PSM, 234 patients were matched in each group, and the baseline characteristics were well balanced between these two groups. As can be seen in **Table 6**, the vast majority of patients was at the regional and distant stages. Using matched data, surgery plus chemotherapy was associated with survival advantages than surgery alone for selected invasive IPMN patients (*P* = 0.0072) (**Figure 4B**).

**Table 6:** Demographic and clinical characteristics of IPMN patients undergoing surgery or surgery plus chemotherapy before and after PSM.

Variables	Before PSM			After PSM		
	Surgery (*n* = 417)	Surgery + chemotherapy (*n* = 280)	*P*-value	Surgery (*n* = 234)	Surgery + chemotherapy (*n* = 234)	*P*-value
Gender			0.747			0.926
Male	236 (56.6%)	155 (55.4%)		125 (53.4%)	124 (53.0%)	
Female	181 (43.4%)	125 (44.6%)		109 (46.6%)	110 (47.0%)	
Age (years)			<**0.001**			0.778
<70	211 (50.6%)	181 (64.6%)		136 (58.1%)	139 (59.4%)	
≥70	206 (49.4%)	99 (35.4%)		98 (41.9%)	95 (40.6%)	
Race			0.172			0.077
Black	43 (10.3%)	18 (6.4%)		32 (13.7%)	17 (7.3%)	
White	338 (81.1%)	233 (83.2%)		178 (76.1%)	191 (81.6%)	
Other	36 (8.6%)	29 (10.4%)		24 (10.2%)	26 (11.1%)	
Tumor size (cm), ± SD	3.70 ± 2.67	4.25 ± 5.94	0.102			
Marital status			0.439			0.056
Married	256 (61.4%)	180 (64.3%)		135 (57.7%)	156 (66.7%)	
Other	161 (38.6%)	100 (35.7%)		99 (42.3%)	78 (33.3%)	
Location			0.988			0.525
Head	307 (73.6%)	206 (73.6%)		171 (73.1%)	177 (75.6%)	
Body/tail	110 (26.4%)	74 (26.4%)		63 (26.9%)	57 (24.4%)	
Grade			<**0.001**			0.929
Well differentiated	237 (56.8%)	178 (63.6%)		150 (64.1%)	147 (62.8%)	
Poorly differentiated	53 (12.7%)	62 (22.1%)		47 (20.1%)	47 (20.1%)	
Unknown	127 (30.5%)	40 (14.3%)		37 (15.8%)	40 (17.1%)	
Tumor number			0.121			1.000
Single	371 (89.0%)	259 (92.5%)		215 (91.9%)	215 (91.9%)	
Multiple	46 (11.0%)	21 (7.5%)		19 (8.1%)	19 (8.1%)	
Tumor stage			<**0.001**			1.000
Localized	192 (46.0%)	57 (20.4%)		57 (24.3%)	57 (24.3%)	
Regional	188 (45.1%)	177 (63.2%)		142 (60.7%)	142 (60.7%)	
Distant	37 (8.9%)	46 (16.4%)		35 (15.0%)	35 (15.0%)	

PSM, propensity score matching; SD, standard deviation. Bold indicates significance.

## Discussion

IPMN and MCN are the most common pancreatic cystic neoplasms. Differences in clinical characteristics and prognosis have not been well investigated in a large-cohort study. The current study demonstrated that the demographic and clinical characteristics were significantly different between invasive IPMN and invasive MCN using the SEER database. Compared with invasive IPMN patients, invasive MCN patients were more likely to be younger, female, with a larger tumor size, and with tumors of the pancreatic body or tail, and patients with invasive IPMN presented at more advanced tumor stages and were associated with worse long-term survival outcomes compared to those with invasive MCN. With respect to the management of invasive IPMN, surgery remained the cornerstone of treatment for localized stage patients, while surgery plus chemotherapy may provide additional survival benefits in selected patients after adjusting for baseline characteristics, especially in patients with advanced stages.

Among the clinical variables, female sex predominance was observed in the invasive MCN cohort, which was in line with previous studies (16, 17), while the incidence rates of invasive IPMN were similar in both sexes. However, the exact cause of gender distribution was not well learned. Further studies are required to fully understand the gender differences of these two diseases.

It is noteworthy that the tumor size in patients with invasive MCN was significantly larger compared to that in patients with invasive IPMN. However, the survival outcomes were even worse in invasive IPMN patients, and the tumor size was not significantly associated with overall survival in invasive MCN patients. The underlying reason for the association between tumor size and prognosis remained unclear. Previous studies suggested that larger MCNs tend to be symptomatic and more indolent. Compared to invasive IPMN, invasive MCN was more commonly associated with a lower aggressive behavior (18).

In survival analysis, we found that the OS and CSS were significantly better in patients with invasive MCN compared to those with invasive IPMN. Interestingly, among patients who were with older age (≥70), male, other race, of tumors in the pancreatic head, and with distant disease, no significant survival differences were observed between invasive IPMN and MCN cohorts. This might be attributed to the limited samples of invasive MCN in these subgroups. Further investigations to account for this discrepancy based on a large sample size are needed to conduct a more specific and detailed subgroup analysis.

On the basis of available guidelines and consensus, surgical resection for resectable and borderline-resectable invasive IPMN patients has been strongly recommended due to the encouraging survival results following surgery (19, 20). In our study, a subgroup analysis of overall survival was performed according to the tumor stage. As shown in the stage-matched survival analysis, patients who only underwent surgery have a more favorable prognosis than those who received other treatments in localized stage. As for the regional stage, surgery plus chemotherapy tended to result in significantly better overall survival. In terms of the patients with invasive MCN, surgery alone was associated with a similar overall survival compared to surgery plus chemotherapy in our study. According to the 2015 AGA and 2017 IAP guidelines (11, 12), surgery was strongly recommended for all MCNs regardless of the tumor diameter, whereas the 2018 European guideline suggested that surgical operations should only be performed in patients with a tumor size larger than 4 cm or with the presence of a nodule. Some studies argued that a conservative management for asymptomatic MCNs was feasible (21). A systematic review including 52 papers was conducted to investigate the natural history and prognosis of pancreatic MCNs, showing that the surveillance of MCNs less than 4 cm in size appeared to be acceptable and safe (22). Our study displayed that more than 85% of MCNs received surgery between 2000 and 2018, and the long-term survival outcomes were satisfactory. Given the small but measurable risk of malignant transformation, the observation strategy should be undertaken with caution. Further high-quality studies are needed to develop the optimal treatment strategies of invasive MCN.

In our large population-based analysis of 2,300 patients with invasive IPMN, multivariate COX regression analysis revealed that surgery was associated with improved overall survival, while chemotherapy was not the independent risk factor for overall survival in the entire study cohort. Several studies have substantiated the prognostic significance of surgical treatment. Unlike pancreatic ductal carcinoma, the impact of chemotherapy in patients with invasive IPMN is still a matter of debate. Within a study including 102 invasive IPMN patients treated between 1990 and 2016, Marchegiani *et al.* demonstrated that adjuvant chemotherapy could improve survival only in patients with positive nodal status and tubular differentiation (23). In another retrospective study on 103 patients collected from 1993 to 2018, Rodrigues *et al.* examined the impact of adjuvant chemotherapy and found that it could not provide survival benefits in node-negative patients but even compromise the prognosis (24). However, a systematic review of 11 studies and 3,393 invasive IPMNs found that node-positive patients could benefit from adjuvant chemotherapy (25). In order to determine the potential role of chemotherapy in the invasive IPMN cohort, we assessed the outcomes between patients who underwent surgery alone and those who received surgery plus chemotherapy after adjusting for confounding by the PSM method. Using matching data, we found that surgery plus chemotherapy could confer survival benefits in invasive IPMN patients with older age and advanced stages. Our findings confirmed that the combination of surgery and chemotherapy would translate into survival benefits for these selected patients.

There were some limitations in our study. Firstly, the current study was limited by the retrospective design. Secondly, some important factors related to long-term survival were lacking, such as patients’ performance status, surgical type and approach, underlying diseases, and comorbidities. Thirdly, disease recurrence and information on treatment schemes were not recorded in the SEER database. Finally, our analysis was based on populations in the United States, which might limit the generalization to other regions.

## Conclusion

In conclusion, our study comprehensively investigated the clinicopathologic features, treatment patterns, and survival outcomes of patients with the two common subtypes of pancreatic cystic neoplasms, invasive IPMN and invasive MCN. While invasive MCN experienced better overall survival compared to invasive IPMN in the subgroups of patients with local–regional disease, the survival advantages disappeared in patients with distant disease. Furthermore, we found that the combination of surgery and chemotherapy was associated with survival benefits in selected invasive IPMN patients, especially those with older age and advanced tumor stages.

## Data availability statement

Publicly available datasets were analyzed in this study.These data can be found here: https://seer.cancer.gov/.

## Ethics statement

The studies involving human participants were reviewed and approved by the Qingdao Municipal Hospital. The current study is based on the SEER database, and the requirements for informed consent were waived off due to the retrospective design.

## Author contributions

Conception and data collection: GS. Drafting and statistical analysis: ZY. All authors contributed to the article and approved the submitted version.

## Funding

This study was supported by the Hepatobiliary and Pancreatic Cancer from Hubei Chen Xiaoping Foundationfor Scientific and Technological Development.(no. CXPJJH11900001-2019206)

## Conflict of interest

The authors declare that the research was conducted in the absence of any commercial or financial relationships that could be construed as a potential conflict of interest.

## Publisher’s note

All claims expressed in this article are solely those of the authors and do not necessarily represent those of their affiliated organizations, or those of the publisher, the editors and the reviewers. Any product that may be evaluated in this article, or claim that may be made by its manufacturer, is not guaranteed or endorsed by the publisher.
